# Mechanical Properties of Lightweight Cementitious Cellular Composites Incorporating Micro-Encapsulated Phase Change Material

**DOI:** 10.3390/ma14247586

**Published:** 2021-12-10

**Authors:** Zixia Wu, Yading Xu, Branko Šavija

**Affiliations:** Microlab, Faculty of Civil Engineering and Geosciences, Delft University of Technology, 2628 CN Delft, The Netherlands; wuzixia2018@163.com (Z.W.); B.Savija@tudelft.nl (B.Š.)

**Keywords:** cementitious materials, architected cellular materials, phase change materials, indirect 3D printing

## Abstract

This work focuses on combining digitally architected cellular structures with cementitious mortar incorporating micro-encapsulated phase change material (mPCM) to fabricated lightweight cementitious cellular composites (LCCCs). Voronoi structures with different randomness are designed for the LCCCs. Aided by the indirect 3D printing technique, the LCCCs were prepared with a reference mortar (REF) and a mortar incorporating mPCM. The compressive behavior of the LCCCs was studied at the age of 28 days, by experimental and numerical methods. It was found that the highly randomized Voronoi structure and the mPCM have minor negative influence on the compressive properties of the LCCCs. The mPCM incorporated LCCCs have high relative compressive strength compared to conventional foam concrete. Furthermore, the critical role of air voids defects on the compressive behavior was identified. The highly randomized porous Voronoi structure, high mPCM content and good compressive strength ensure the LCCCs’ great potential as a novel thermal insulation construction material.

## 1. Introduction

In recent decades, there has been a dramatic increase in energy consumption around the world. Due to the significant growth of population and the proliferating housing demands, this increase is expected to continue for the next couple of decades. In order to alleviate the burden of energy consumption, thermal insulation materials are widely adopted in residential and commercial buildings. The excessive electricity consumption for maintaining comfortable indoor temperature can be saved and the corresponding carbon emission reduced by applying these materials to optimize the energy efficiency of building heating and cooling systems [1,2,3].

The possibility of construction materials with good thermal insulation properties is an imperative driving force for the researchers to design novel materials with low thermal conductivity while maintaining reasonable mechanical properties. As the most used construction materials, cementitious materials have been investigated by many studies for the purpose of insulation. Foam concrete is a commonly studied lightweight cementitious material. The density of foam concrete ranges usually from 300 to 1800 kg/m^3^, with a corresponding low thermal conductivity (0.1–0.7 W/m·K), making it a good choice to be used as thermal insulation construction material [4,5,6]. Similarly, some researchers focus on adding lightweight ingredients or introduce cavities to achieve high porosity in the concrete to modify the thermal properties. Ruiz-Herrero et al. [7] tried to decrease the thermal conductivity of concrete by including 5% plastic waste aggregate. Bouguerra et al. [8] proved experimentally that adding wood aggregate into clayey concrete can improve the thermal performance. Marais et al. [9] used 3D printing to fabricate foam concrete cavity wall and proved its enhanced thermal performances compared to a solid concrete wall.

**Figure 1:**
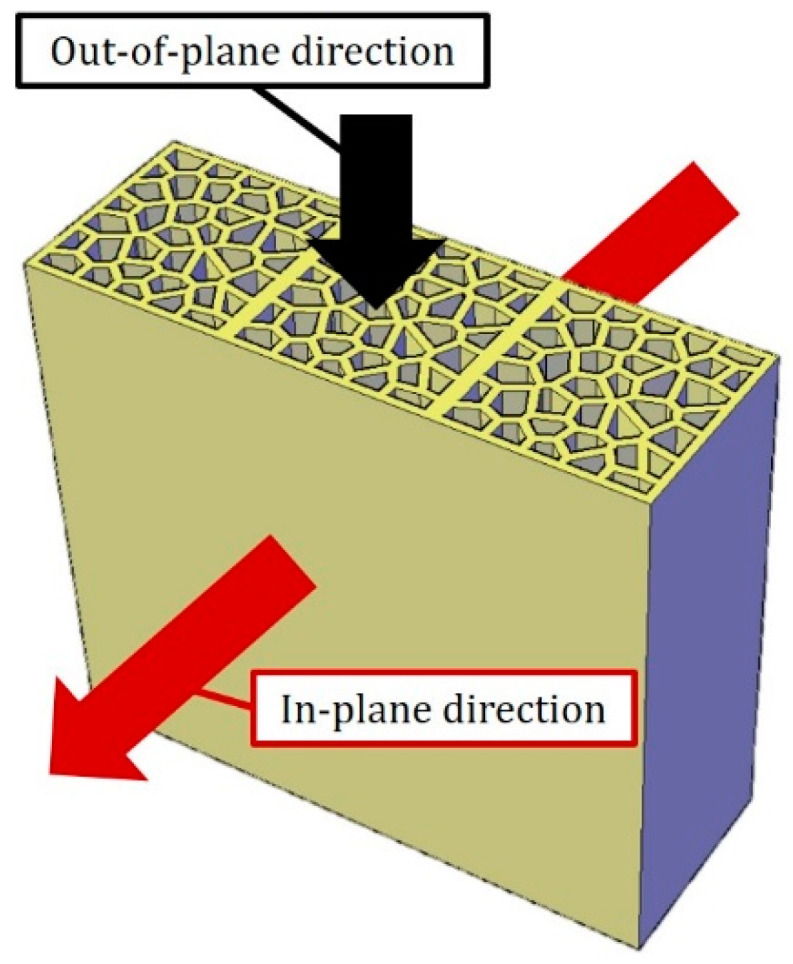
Schematics of a wall built by LCCCs, the in-plane direction is used for thermal insulation, and self-weight is generated at the out-of-plane direction.

Apart from these approaches, owing to the rapid development of digital fabrication, architected cementitious materials with controlled cellular structures high porosity have attracted research interest recently. These cellular structures such as bio-inspired structures [9,10], lattices [11,12,13], periodic minimal surface structures [11,14] and functional designs [15,16,17] constituted by cementitious materials exhibit good mechanical properties accompanied by high porosity. Architected cellular material ensures the possibility of independently optimizing the cellular structure as well as the constituent material. In terms of thermal insulation, Voronoi structure has been observed to possess excellent properties. Compared to traditional hexagonal honeycombs, References [18,19] demonstrated that Voronoi structure with higher irregularity possess improved thermal insulation property (lower thermal conductivity). With the help of experimental and numerical analysis, many studies have attempted to integrate Voronoi structure constituted by metal and polymers to fabricate cellular composites with excellent thermal performance [19,20,21]. Considering the application of building thermal insulation, 2D Voronoi structure are easy to be manufactured, and the in-plane direction (shown in Figure 1) of the Voronoi structure would be used to insulate heat for the superior thermal properties. Then, the out-of-plane direction naturally becomes the self-weight-bearing direction to withstand compressive gravity load. Although they are not used for structural load bearing purposes, the Voronoi structures still need to have enough stiffness and strength to withstand their self-weight when used for building insulations. However, to the authors’ knowledge, a study focusing on the compressive behavior of cementitious Voronoi honeycombs has not been reported yet. Therefore, while possessing great application potential, the knowledge of the physical and mechanical properties of cementitious materials with Voronoi structures is scarce and worth further investigation.

Another opportunity for improving the performance of cementitious materials to optimize building energy efficiency—the use of phase change materials (PCM) as additives—has been widely explored in recent years. In such applications, the phase change process occurs due to the indoor/outdoor temperature variation, in which the heat is absorbed by melting or is released by solidifying during the liquid–solid transition of the PCM, therefore providing thermal inertia against the temperature fluctuation and therefore increasing the energy storage and efficiency [22,23]. In the last 2 decades, appreciable studies have strived to incorporate the PCMs into the cementitious materials to enhance the energy saving in building environment [24,25,26,27]. However, it has been shown that the addition of PCMs [28,29] causes a decrease in compressive strength and workability of cementitious composites [30,31,32,33].

The concept of combining architected cellular structure with PCMs might be promising for fabricating lightweight cementitious cellular composites (LCCCs) with good mechanical and thermal performance. However, the effect of geometry traits and PCMs on the mechanical behavior of the LCCCs has yet to be fully understood. Therefore, this study aims to elaborate on the mechanical properties of LCCCs with Voronoi structures containing micro-encapsulated phase change material (mPCM). In order to study the effect of Voronoi structural randomness on the compressive behavior, specimens with three different randomness were designed. Using the so-called “indirect” 3D printing technique, the designed LCCCs were prepared and subjected to the uniaxial compression test. The next part dealt with the analysis of the compressive behavior of the LCCCs by experimental results accompanied by numerical simulations. Based on the analysis, the influence of Voronoi randomness and mPCM on the compressive properties of the LCCCs are discussed, and the compressive damage mechanism of the LCCCs is clarified.

## 2. Materials and Methods

### 2.1. Voronoi Geometry Generation

Voronoi tessellation is defined as the domain partition in the m dimensional space consisting of n distinct regions according to [34,35]. Each region, *R_k_*, referred to as the Voronoi cell, is generated by corresponding point *p_k_*, called seed. The Voronoi cell *R_k_* represents the subset of the domain in which the distance of any point to the correspond seed *p_k_* is always closer than its distance to other seed. Mathematically, this can be expressed as:

(1) $$R_{k}=\{x\;in\;X:d\left(x,p_{k}\right)\leq d\left(x,p_{j}\right)\;for\;all\;j\neq k$$

**Figure 2:**
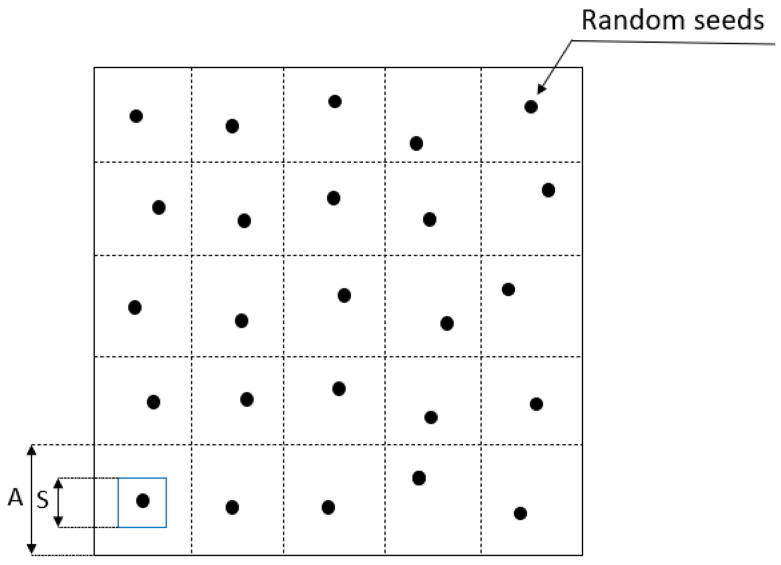
The distribution of random seeds in the square domain.

**Figure 3:**
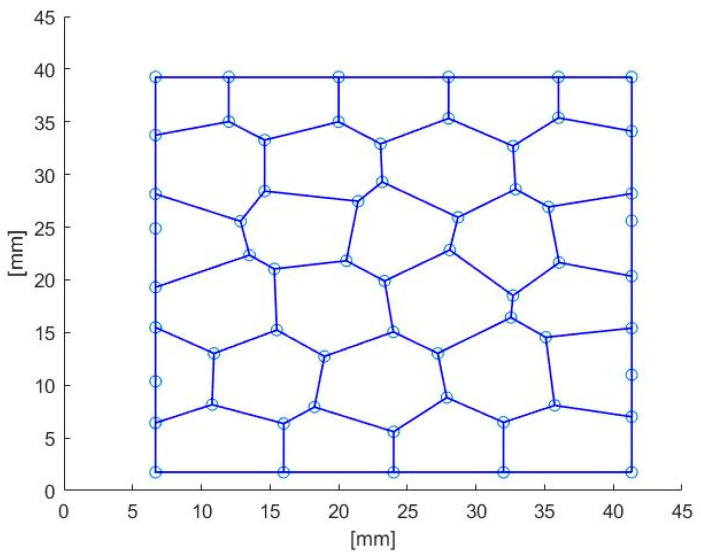
The Voronoi tessellation generated in MATLAB.

In the present research, 2D Voronoi tessellation is employed in MATLAB R2020a. In order to define the location of the randomly scattered Voronoi diagram seeds, a 2D domain was meshed with n × n square cells (shown in Figure 2). In each cell, a single seed point was located. The blue square in Figure 2. indicates the area where the seeds could be located, and the randomness depends on the ratio of blue square and the square cell. The randomness increases while the blue square become larger. The randomness was used to present the irregularity of structure. In order to determine the coordinate of points, the randomness δ = S/A was applied in MATLAB code. Theoretically, δ can be in the range from 0 to 1; the higher the δ, the more irregular the Voronoi geometry becomes. The generated points were then used as the seeds of the Voronoi partition. The function Voronoi (x, y) of MATLAB was applied to generate the Voronoi tessellation. Subsequently, the output of the code contains the coordinates of the vertices delimiting polygons associated with the seeds (Figure 3).

In this research, Voronoi structures with 3 different randomness values—0, 0.3, and 0.7—were designed to investigate the effect of irregularity on the mechanical properties of cementitious cellular material. The process of mechanical test and numerical simulation is elaborated in Section 2 and Section 3.

### 2.2. Materials and Mixtures

Prior to designing the mixture proportions of the cementitious materials, the requirements for the mixtures had to be verified. Higher mPCM content was preferred for thermal properties; by contrast, lower mPCM content was preferred to achieve better mechanical properties and workability. A mixture balancing these requirements needed to be developed first.

The mPCM used in this work was composed paraffin wax as phase change core which is encapsulated in a melamine formaldehyde (MF) shell. The same mPCM used in [36] was also used in this study. According to the data provided from manufacturer, the core-to-shell mass ratio was approximately 11.8. The latent heat of fusion of the used mPCM was 143.5 J/g, and the median particle size was 22.53 µm. It is known from [36], by incorporating 20% by volume of the same type mPCM used in this study, the 28 days compressive strength significantly decreased around 50%. In addition, as in this study the specimens were prepared in the same way as reported in [17,37] (explained later), and highly fluid mixtures were of great help in filling the molds. This was paramount for the quality of the hardened samples after 28 days curing. Therefore, based on a reference mix design (REF) adopted from [38,39], the mixture of the mPCM incorporated mortar was designed such that it had a high volume ratio (14.4%) of mPCM meanwhile the same slump to the REF mix was maintained. The mixture designs are listed in the Table 1, and w/b indicates water to binder ratio.

**Figure 4:**
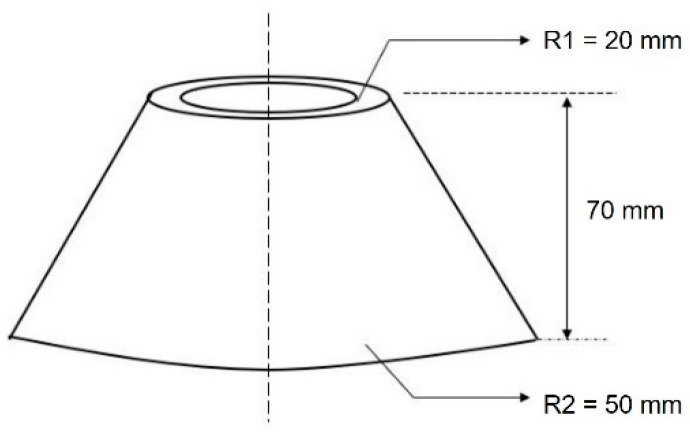
Schematics of the mini slump cone.

**Figure 5:**
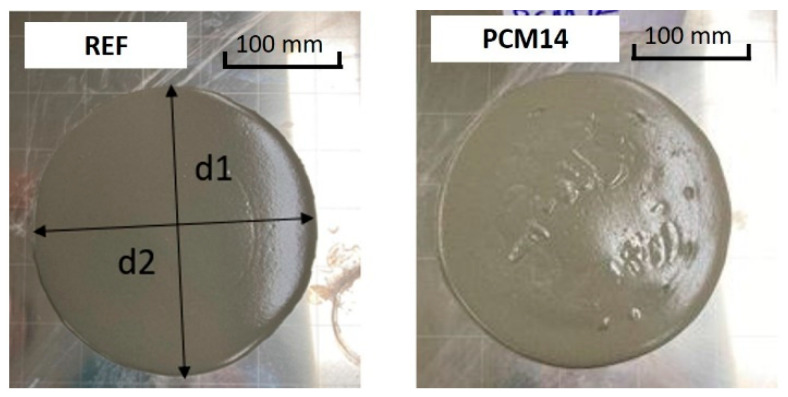
The slump flow of REF and PCM14.

The mixture workability was tested using a “mini slump” cone [40] which characterizes the workability by the spread of mortar [41,42]. The dimensional details of the cone are shown in Figure 4. Freshly mixed mortar was filled into the cone from the top opening which was placed on a glass plate. After 1 min of compacting, the cone was lifted rapidly for the remaining clear mortar to flow as well as to avoid extra inertia on the mortar. After waiting for 30 s, a digital image on the top view of the mortar area was taken (Figure 5). The mortar flowing spread was measured precisely using the average value of the orthogonal diameter by digital image pixels.

### 2.3. Specimen Preparation

**Figure 6:**
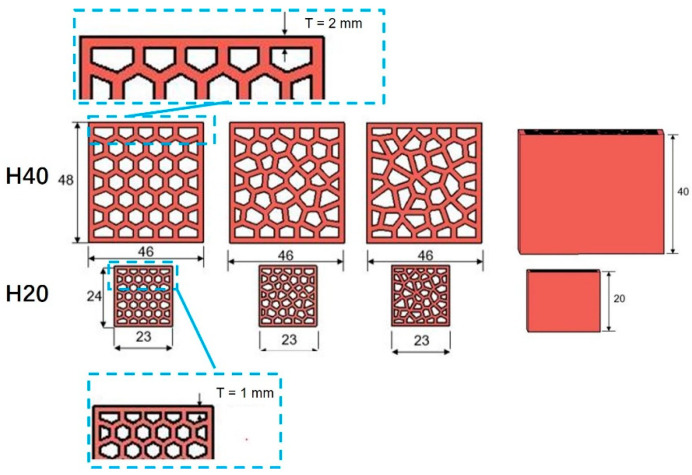
Specimen dimensions and various designs, indicated in [mm].

**Figure 7:**
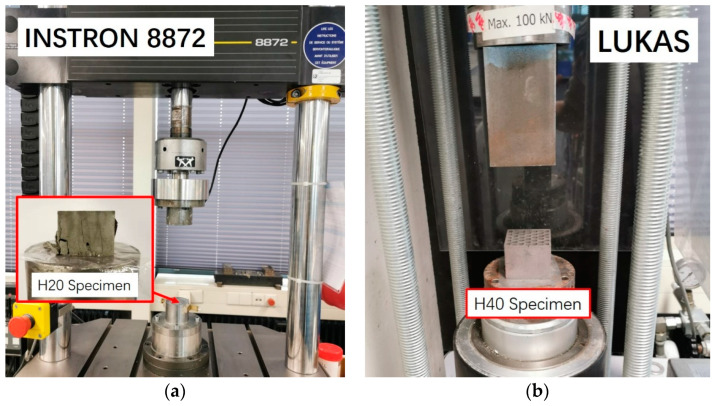
Two different hydraulic press machines, (**a**) INSTRON 8872 for H20 and (**b**) LUKAS for H40.

The cellular structure of the LCCCs fabricated herein is architected by varying the randomness of the Voronoi geometry. Normally, besides the irregularity, the mechanical properties of cellular materials are also influenced by the cellular porosity (relative density) [43,44]. Therefore, the specimen design employed herein was performed under the premise of keeping a constant porosity (the deviation should be smaller than 5%) for different designs. Two sizes of specimens were manufactured in this study as shown in Figure 6: specimens with the size of 48 × 46 × 40 mm^3^ (length × width × height), referred to as H40; and specimens with the size of 24 × 23 × 20 mm^3^ (length × width × height), referred to as H20. Aiming at normalizing the compressive strength of the tested sample, the H40 specimens were designed with a size similar to that defined by a Dutch standard for determination of cement strength (NEN-EN 196-1). On the other hand, the H20 specimens were fabricated with the aim of obtaining the insight of compressive behavior of the cementitious cellular materials. A smaller size was required because the compressive strength of the H40 design was beyond the capacity of the hydraulic testing machine (INSTRON 8872, see Figure 7a) which needed to be used in order to measure the complete compressive stress–strain response, including the post-peak behavior. The H40 specimens were only used to test compressive strength of the LCCCs by a load-controlled hydraulic press LUKAS (Figure 7b). The characteristics of two groups of specimens are given in the Table 2 and Figure 6. The randomness and porosity of H40 and H20 specimens were the same, and the cell wall thickness of H40 and H20 was 2 and 1 mm, respectively.

The specimens were fabricated employing a so-called “indirect 3D printing” workflow proposed in [17] first for fabricating cementitious cellular composites. The same procedure is followed herein and is described as follows:

- In the first step, the ABS (acrylonitrile butadiene styrene) mold was fabricated using fused deposition modeling (FDM) method by employing a commercial 3D printer (Ultimaker 2+). The ABS was heated up until 260 °C, becoming semi-liquid, and then extruded with a nozzle diameter of 0.8 mm. Each filament layer was 0.2 mm high and the printing speed 40 mm/s.
- After 3D printing, the ABS mold was glued to the bottom of a cardboard box. A mixture of two component silicone rubber (consisting of Poly-Sil PS 8510, mixed 1:1 by weight) was poured into the ABS mold. The mold was then put into a vacuum chamber for extracting the air from silicone rubber. After 2 h, the silicone rubber mold had hardened.
- The hardened silicone rubber mold was easily detached from the initial ABS mold. It should be noted that the printed ABS mold and the detached silicone rubber mold are completely reusable. The fresh mortar was poured into the silicone rubber mold and compacted. After 2 days, the cementitious cellular specimens were demolded and cured under water until testing at 28 days.

**Figure 8:**
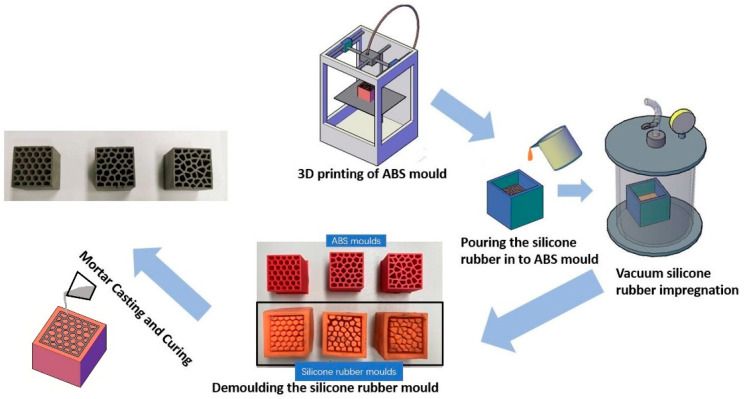
Specimen fabrication working flow of the indirect 3D printing method.

**Figure 9:**
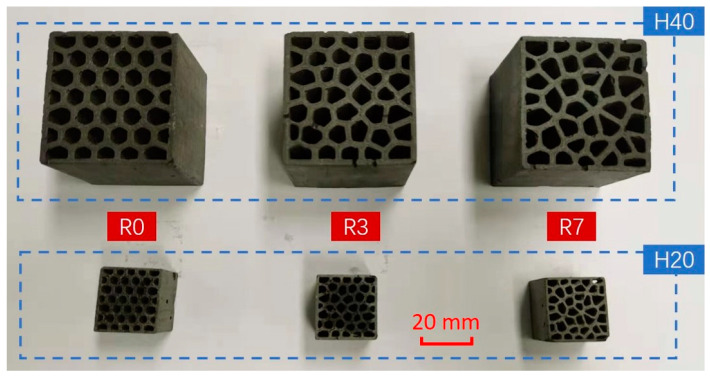
Casted cementitious cellular specimens.

The details of the specimen preparation working flow are given in Figure 8. In addition to cellular samples displayed in Figure 9, REF (see Table 1) and PCM14 (see Table 1) mortar specimens were casted into cube for compressive strength test. These results were also needed as input for the numerical simulations.

### 2.4. Uniaxial Compression Tests

In order to evaluate the compressive behavior of the LCCCs, displacement-controlled uniaxial compressive tests were conducted by a INSTRON 8872 servo hydraulic press (Figure 7a) with a rate of 0.003 mm/s. As discussed by van Mier [45], in the case of cementitious material which cracks rapidly, a very fast response time of the loading system was required, and then the size of specimens could be reduced in order to properly measure strains. This is one of the reasons two specimen sizes were used in this study, as discussed in the previous section.

**Figure 10:**
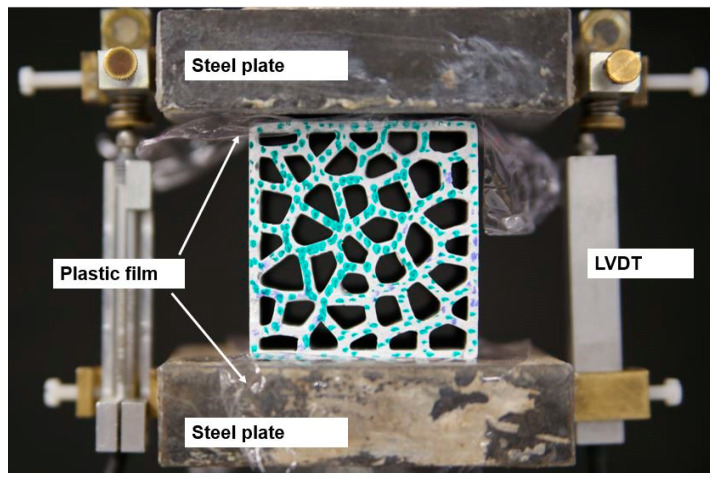
In-plane uniaxial compression test set-up on INSTRON 8872.

The uniaxial compression experiment configuration is shown in Figure 10. The specimen was placed between two steel plates; plastic films were utilized to reduce friction between the loading plates and the specimen. Linear variable displacement transducers (LVDTs) were installed on the side face of the steel plates for recording the displacement. Three duplicates were tested for all specimens.

## 3. Numerical Simulations

### 3.1. Concrete Damage Plasticity (CDP) Model

In this research, numerical simulations were carried out by employing Commercial Software Abaqus/Explicit. The principle of analyzing damage process of the cementitious materials was based on the concrete damage plasticity model [46,47]. The CDP model considers the effect of plasticity damage on the compressive and tensile response of the material, and the formulation is as expressed in Equations (2)–(7). By conducting the compression test on cementitious cubic specimen and bending test on cementitious prismatic specimen, the initial elastic modulus *E*_0_, compressive inelastic strain $\varepsilon_{c}{}^{in,h}$ and tensile cracking strain $\varepsilon_{t}{}^{ck,h}$ are obtained. $\varepsilon_{c}{}^{pl,h}$, $\varepsilon_{t}{}^{pl,h}$ are compressive plastic strain and tensile plastic strain, which are key parameters for controlling the compressive and tensile failure. They are derived from $\varepsilon_{c}{}^{in,h}$ and $\varepsilon_{t}{}^{ck,h}$ and their corresponding damage parameter *d_c_*, *d_t_.* The compressive ($d_{c}$) and tensile ($d_{t}$) damage parameter represent the elastic modulus reduction, which is calculated based on the ratio of nominal stress ($\sigma_{c}\;in\;compression,\;\sigma_{t}\;in\;tension$) to the compressive $\sigma_{cu}$ and tensile $\sigma_{tu}$ ultimate strength, respectively.

(2) $$d_{c}=1-\frac{\sigma_{c}}{\sigma_{cu}}$$

(3) $$d_{t}=1-\frac{\sigma_{t}}{\sigma_{tu}}$$

The CDP model is calibrated by these parameters which are denoted as the compressive behavior and tensile behavior input of cementitious material.

(4) $$\sigma_{c}=\left(1-d_{c}\right)\cdot E_{0}\cdot \left(\varepsilon -\varepsilon_{c}{}^{pl,h}\right)\;$$

(5) $$\sigma_{t}=\left(1-d_{t}\right)\cdot E_{0}\cdot \left(\varepsilon -\varepsilon_{t}{}^{pl,h}\right)$$

(6) $$\varepsilon_{c}{}^{pl,h}=\varepsilon_{c}{}^{in,h}-\frac{d_{c}}{\left(1-d_{c}\right)}\cdot \frac{\sigma_{0}}{E_{0}}\;$$

(7) $$\varepsilon_{t}{}^{pl,h}=\varepsilon_{t}{}^{ck,h}-\frac{d_{t}}{\left(1-d_{t}\right)}\cdot \frac{\sigma_{0}}{E_{0}}$$

### 3.2. Model Calibration

**Figure 11:**
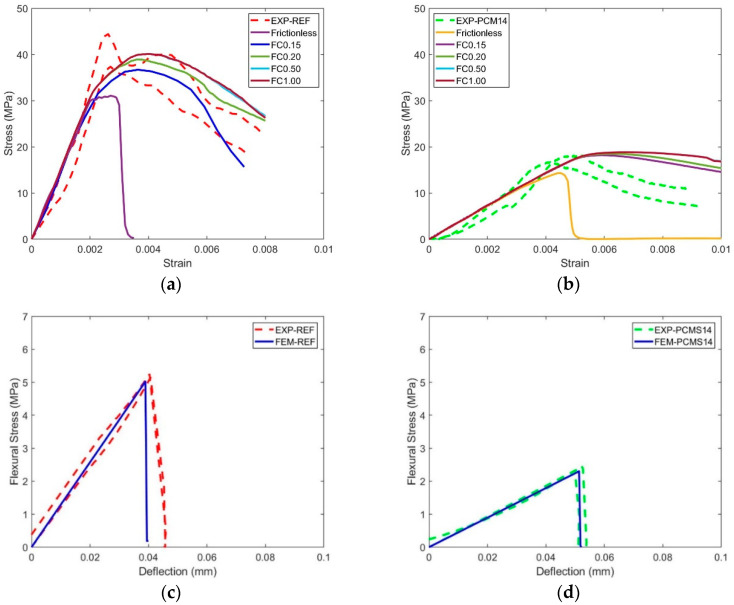
Comparison between experiment and calibration simulation results, (**a**) compression results of REF, (**b**) compression results of PCM14, (**c**) bending results of REF and (**d**) bending results of PCM14; the dashed lines indicate experimental measurement uncertainties.

To calibrate the model input parameters, numerical uniaxial compressive and tensile tests were performed on the cubic and prismatic specimens. As CDP is intrinsically mesh size dependent, 1 mm tetrahedron linear mesh (C3D4) was used for all simulations. The loading rate was maintained as 0.003 mm/s, which is consistent with the experiment loading condition. Compressive input and tensile parameters (presented in Table 3, Table 4, Table 5 and Table 6) were obtained by a trial-and-error method to fit experimentally obtained stress–strain and flexural-deflection curves. The plasticity parameters of the CDP are shown in Table 7, for both the REF and PCM14 mixture, and the same input was used. The boundary conditions of the numerical uniaxial compressive and bending tests are the same as in the experiments: in compression, two rigid body plates were generated to apply the external load on the specimens, and the influence of friction coefficient was studied while performing the uniaxial compression tests. A friction coefficient was varied from 0 (frictionless) to 1 for the contact between the rigid plates and the specimen top and bottom surfaces. In bending, four loading rollers were used to apply the external load, a same friction coefficient 0.15 was adopted from [39], which has a similar four-point-bending loading scheme. The dimension of the specimen for uniaxial compression calibration test was 20 × 20 × 20 mm^3^, and 40 × 10 × 140 mm^3^ for four-point bending test. All degrees of freedom (DOFs) of each rigid plate/loading roller were constrained on a reference point at the center of the plate/roller. Vertical downward displacement was applied on the reference point on the top while other DOFs of the reference point were completely fixed. All DOFs of the reference point on the bottom plate were completely fixed. It can be found in Figure 11 that, in general, the simulated uniaxial compressive and flexural curves show good agreement with experimental curves, which allows the use of calibrated input parameters in further simulations. Regarding the uniaxial compression, the influence of friction coefficient (FC) on the compressive behavior of the cubic specimens is quite obvious. By increasing the FC from frictionless (FC = 0) to 0.15, the compressive strength substantially increased, especially for the REF. The influence of FC on the softening branch is even more significant. After the FC was increased to 0.15, the dependence of compressive strength on the FC becomes very minor. More detailed and in-depth discussion on the role frictional contact in the simulation of the LCCCs is given in Section 4.4 and Section 4.5.

### 3.3. Simulations of the Cellular Specimens

At the macroscale, which is the scale of this study, cementitious mortar is usually treated as homogenous material. While, when defects are present, they need to be specifically considered because they may cause stress concentration and substantially influence mechanical properties, normally, a certain amount of air voids defect (entrained or entrapped) exists in hardened cementitious mortar, especially at the surface of a specimen in contact with the mold surface. In the present study, due to the complex geometry of the specimen and the mold, there is much large surface area in contact with the silicon molds compared to conventional solid concrete specimens without the cellular structures, for example, concrete cubes. Therefore, compared to regular specimens, there might be a higher chance that air bubbles agglomerate on the mold surface after casting and form additional air void defects in the hardened specimens. Using CT scan to provide reliable input for the numerical model is an option; however, the data size to be processed for each computational specimen is (still) prohibitive. Furthermore, for reliable results, usually several specimens need to be scanned and analyzed, which causes extremely heavy computing requirements. Therefore, herein, a simplified approach of generating the numerical model of the tested specimens is used in this study.

**Figure 12:**
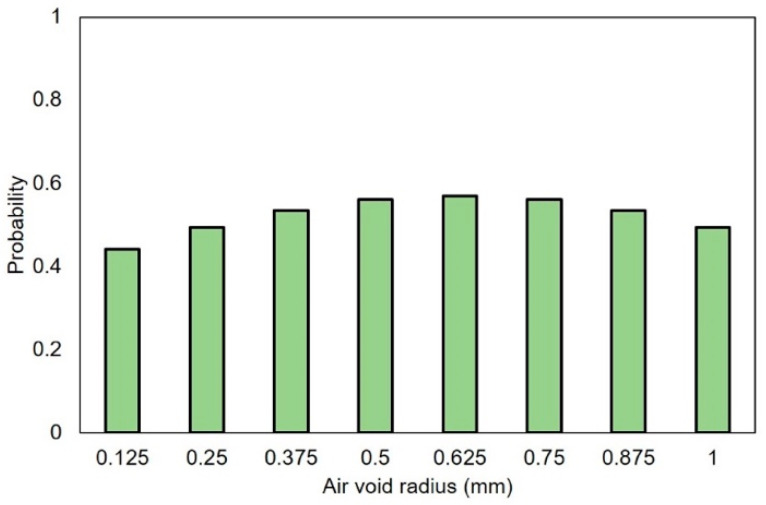
Distribution of air void radius.

**Figure 13:**
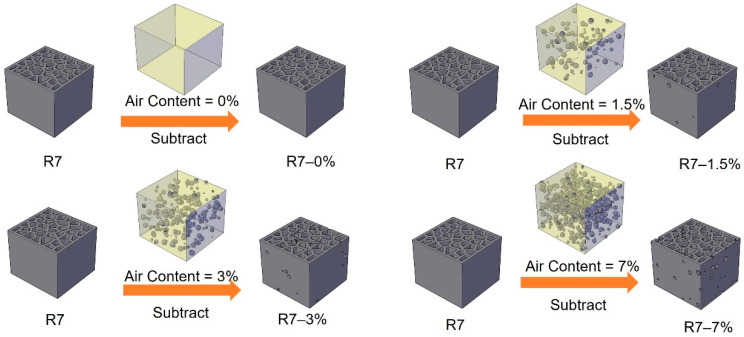
Schematics of generating cellular specimens with virtual air void defect structure.

According to [48,49,50], the air void content of cementitious materials is around 1%–6%, and the radius of air voids is commonly reported to mainly distribute from micrometers up to several millimeters. In order to study the influence of air voids, in this study, four air content values are used: 0%, 1.5%, 3% and 7%. Very small air voids require very dense mesh in the simulation, and they need extremely high computing effort. For simplification, spheres with radius ranging from 0.125 to 1 mm, which are commonly found in cementitious mortar, are used to generate virtual air void structure of the cementitious mortar. Similar normal size distribution of this range reported in [51] was used for the generated spheres. The size distribution of the generated air voids is shown in Figure 12. The specimen generating process is shown in Figure 13; at first the coordinates of the sphere center were randomly generated within the dimension of 24 × 23 × 20 mm^3^ (same size of the H20 specimens), then the spheres were subtracted from the H20 specimens such that H20 specimens with different air void structures are generated. All specimen models were generated by AutoCAD 2019 and then imported to Abaqus/Explicit. The total number of C3D4 elements of R7–0%, R7–1.5%, R7–3% and R7–7% is 57,713, 61,955, 89,605 and 187,415, respectively.

**Figure 14:**
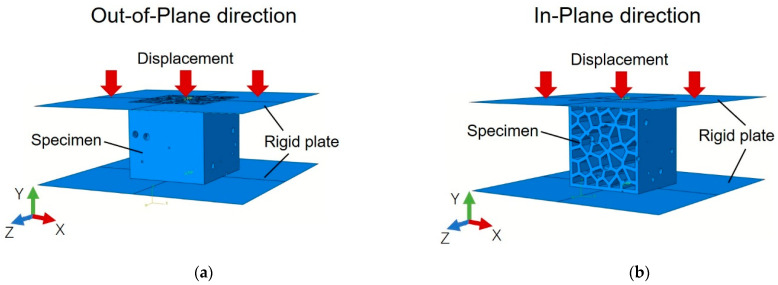
Loading setup in the simulation of (**a**) the out-of-plane direction and (**b**) the in-plane direction.

As shown in Figure 14, the simulation on the cellular specimens is performed at the out-of-plane and in-plane directions. At the in-plane direction, frictionless contact was used. At the out-of-plane direction, due to the small contact area between the plate element of the specimen and the steel loading plates, the specimens would show unrealistic slide under frictionless condition. Therefore, similar to the method used in [39], a minor friction coefficient 0.15 is used to avoid unrealistic sliding. The influence of friction coefficient is discussed in detail in Section 4.4. A mass scaling was applied in the simulations, and the energy balance is reached. For all simulations, the total energy (should be as close to zero as possible) maintained lower than 5 × 10^−4^ mJ; the kinetic energy is lower than 3% of the internal energy; the final stable time increment is lower than 4 × 10^−3^.

## 4. Results and Discussion

### 4.1. Influence of Voronoi Randomness on Out-of-Plane Strength

Compressive strength of cementitious material is one of the most important factors determining the capability of damage resistance, and it ought to be measured by standard testing. Thus, as mentioned in Section 2.4, H40 specimens were used in the mechanical test for measuring the compressive strength.

**Figure 15:**
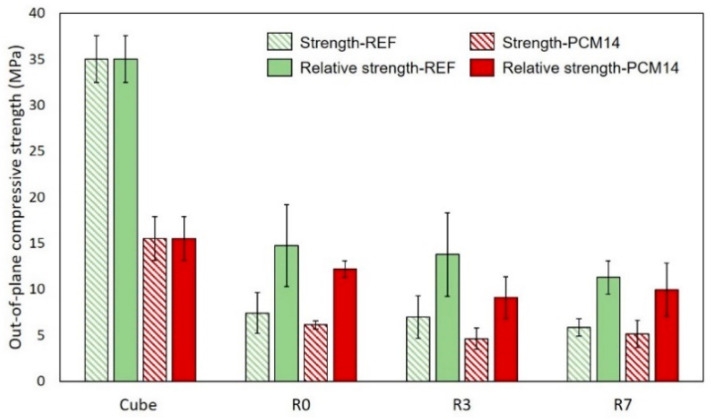
Comparison of the out-of-plane compressive strength between R0, R3 and R7 (measured from H40 specimens) and the material cube strength.

As shown in Figure 15, the out-of-plane compressive strength of all cellular specimens is much lower than the material cube strength, as expected for specimens with such high porosity. Comparing to the regular honeycomb R0, specimens with a randomized Voronoi structure (R3 and R7) show relatively lower compressive strength. For the REF specimens, compressive strength of R0, R3 and R7 is 7.4, 7.0 and 5.8 MPa, respectively.

Considering the high porosity of the cellular specimens, in order to provide fairer comparison, the compressive strength of the cellular specimens is normalized by their porosities to the same volume of the cube specimens, defined as relative strength. The relative strength of the cellular specimens is also provided in Figure 15. For the REF specimens, R0, R3 and R7 have a relative strength of 14.7, 13.7 and 11.3 MPa, respectively. Even normalized by the porosity, the strength of the cellular specimens still significantly decreased by approximately 60% comparing to the material cube strength. This may be attributed to the significant influence of defects or imperfections on the compressive behavior of the cellular materials. A more in-depth mechanism is elaborated later in Section 4.4, by analyzing numerical results and experimental measurements of the H20 specimens.

### 4.2. Influence of mPCM on Out-of-Plane Strength

Although the mPCM can improve thermal performances of cementitious materials, the negative effect on mechanical property is found in [25,29,31] to be the main drawback of incorporating mPCM in cementitious mixture. The mPCM are encapsulated by a polymeric shell which is easily broken or deformed during the mixing procedure [29]. Porosity increases due to the presence of broken or deformed capsules [25], while leaked paraffin mixes with cement particle and hinders hydration process. Therefore, integrating mPCM in cementitious materials normally leads to obvious compressive strength decrease. Depending on the mixture design, adding around 14% of mPCM by volume results in a compressive strength loss of 20–60% [23]. This is consistent with the experimental results obtained in this study: the cube compressive strength of PCM14 is 15.5 MPa, which is 55.7% less than the cube strength of REF (see Figure 15). However, for the cellular specimens, the strength reduction caused by mPCM is much less significant. The compressive strength of R0, R3 and R7 specimens made of PCM14 mixture only decreased by 17.2%, 34.0% and 12.1% compared to the REF group counterparts, respectively. This means that the negative influence of mPCM on the compressive strength of cementitious materials would not be a drawback for the LCCCs.

As mentioned before, higher Voronoi randomness ensures better thermal insulation property [18,19]. Meanwhile, in terms of compressive strength, the R7-PCM14 also did not show obvious strength decrease compared to the R7-REF. In this sense, combining highly randomized Voronoi structure with mPCM to fabricate LCCCs for thermal insulation purpose would be a very promising strategy. Among all studied LCCCs in this work, R7-PCM14 is the optimal choice.

### 4.3. Comparison of the LCCCs to Foam Concrete

**Figure 16:**
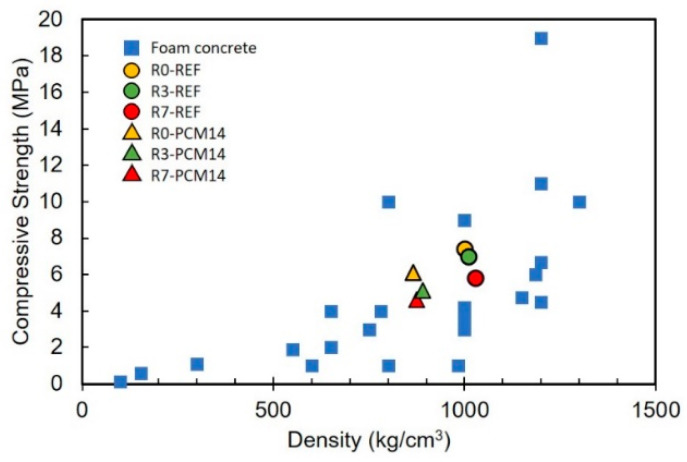
Comparison of the compressive strength of the cellular specimens with normal foam concrete [8,10].

Considering the thermal and mechanical properties of the LCCCs, in engineering practice, the LCCCs would be used as lightweight non-structural elements, for instance façade panels. Therefore, the role of the mPCM incorporated LCCCs in engineering practice resembles conventional foam concrete. The compressive strength comparison between that of foam concrete and the fabricated LCCCs is shown in Figure 16. The out-of-plane compressive strength of the LCCCs made of PCM14 is similar to the foam concrete with similar density (porosity). In a recently reported case when mPCM is combined with foam concrete [52], the specific compressive strength (compressive strength divided by density) is less than 2 KPa·m^3^/kg which is also much lower than the 4.4 KPa·m^3^/kg of the fabricated R7-PCM14. Moreover, according to [53], the thermal conductivity of the mPCM incorporated LCCCs is 0.35 W/m·K which is similar to the value of foam concrete at a density of 1000 kg/m^3^. In some reported cases, the compressive strength of such foam concrete reaches 8–10 MPa. In order to further improve the compressive strength of the LCCCs, the determinative factors are discussed in detail in the next section.

### 4.4. Impact of Air Void Defects on Compressive Behavior

**Figure 17:**
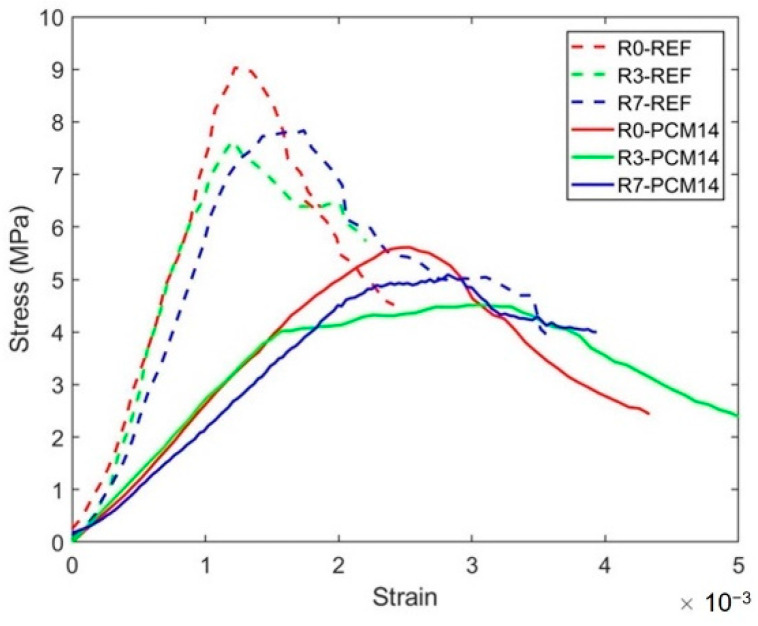
Stress–strain curves of the LCCCs on the out-of-plane direction obtained both from the REF specimens and PCM14 specimens, measured by H20 specimens.

Due to the testing set-up limitation mentioned in Section 2.4, the H20 specimen was used to study the compressive behavior and clarify the compressive damage mechanism of the LCCCs. It is observed in Figure 17 that, at the out-of-plane direction, the stress–strain curves of the lightweight LCCCs resembles the compressive response of conventional cementitious materials: for both REF and PCM14 specimens, as soon as the external was applied, an ascending branch can be observed, which indicates the elastic deformation stage. Afterward, the slope started to flatten and followed by a peak stress of the specimen, and then a softening branch occurs representing the process of post-peak.

**Figure 18:**
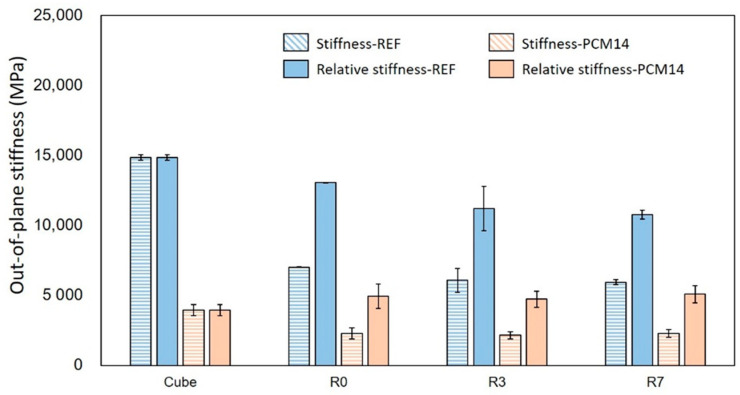
Comparison of the out-of-plane stiffness between R0, R3 and R7 and the material cube stiffness (E-modulus).

Within the elastic stage, the stiffness/E-modulus of the specimens is obtained by measuring the slope of all specimens up to 0.3σ_peak_. The value measured from the cubic specimens is regarded as the material E-modulus, while the values measured from the LCCCs are comparatively regarded as the out-of-plane stiffness of the corresponding specimens. Due to the high porosity, the stiffness of all tested LCCCs on the out-of-plane direction has strikingly reduced to approximately 50% of the material E-modulus, shown in Figure 18. Nevertheless, when the E-modulus of all tested specimen is normalized by their porosity (namely the relative E-modulus), the discrepancy between the material E-modulus and the stiffness of the LCCCs becomes much smaller. For LCCCs made of REF mixture, the stiffness of R0, R3 and R7 only dropped 12%, 24% and 27% respectively. Surprisingly, for the LCCCs made of PCM14, the relative stiffness is even higher than the material E-modulus.

After the elastic regime of the stress–strain curves (see Figure 17), a peak stress is reached for all test groups. It is worth noticing that the peak stress measured from H20 specimen is slightly higher than the compressive strength measured from H40 displayed in the Figure 15. It is plausible that the size effect [54] has influence concerning the peak stress which means the smaller specimens tend to achieve a higher compressive strength. This peak load indicates the compressive strength of the LCCCs, and it was found to be substantially determined by the air void defects.

**Figure 19:**
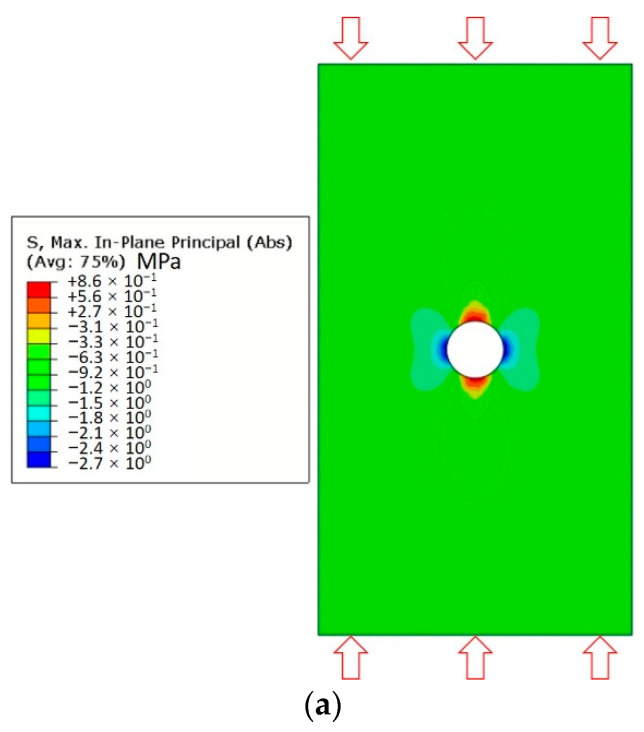

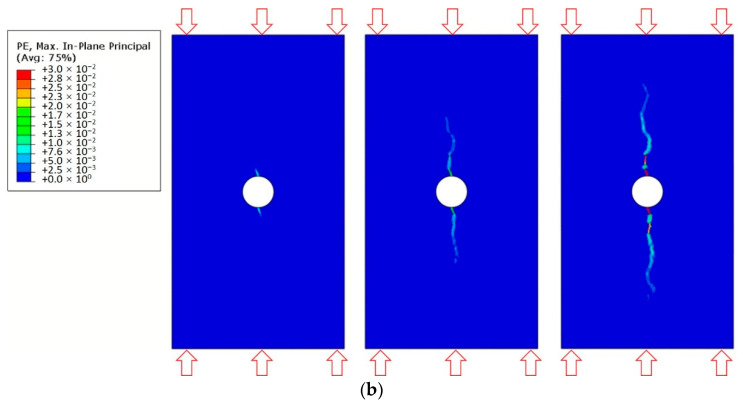
Simulated (**a**) stress concentration at the edge of a circular hole on a compressive loaded plate and (**b**) crack initiation and propagation process on a compressive loaded plate.

Considering the geometrical features of the LCCCs, at the out-of-plane direction, they can be seen as a multiple thin-walled structure consisted by plate elements with various orientations. When loaded from the out-of-plane direction, the deformation and fracture behavior of each plate determine the global behavior of the cellular specimen. From the theory of fracture mechanics, when an external compressive load applied on top of an infinite plate, due to the structural discontinuity intensive compressive and tensile stress, it concentrates at the edge of the circular hole. In theory [55], three times the compressive stress appears at the direction perpendicular to the applied stress, and the tensile stress concentrates at the parallel direction to the applied stress with the same absolute value. This was also simulated using a FEM tool, as shown in Figure 19a when 1 MPa compressive load was applied on top of a plate. Tensile and compressive stress concentrates at the edge of the circular hole. Note that in the FEM simulation the plate is not an infinite plate, such that the compressive and tensile stress are not exactly theoretical values.

**Figure 20:**
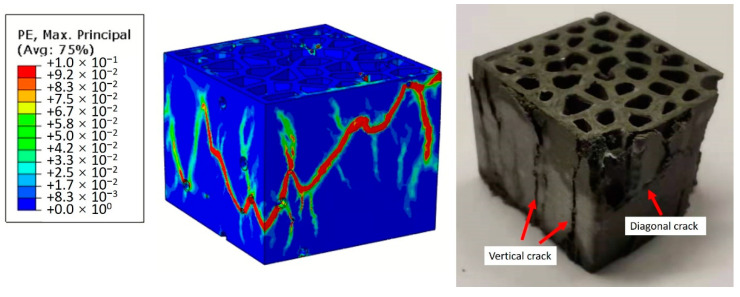
Comparison of the crack pattern obtained from simulated and experimental tested specimens, obtained from R7-PCM14.

For cementitious materials, the tensile strength is significantly lower than their compressive strength. In this work, since the compressive strength of PCM14 and REF are approximately 8 times higher than their tensile strength, cracks easily initiate around the air voids at the location with high tensile stress concentration. After initiation, the crack propagates parallel to the direction of the applied load, forming a vertical cracking along the plate (see Figure 19). These vertical cracks were also observed from the experiments and simulations of the LCCCs (see Figure 20). In this sense, the strength of those plate elements with air voids is significantly reduced, and these plate elements become critical parts of the LCCCs, which eventually results in reduction of the compressive strength than the case without any introduced air voids. More air void defects lead to more critical parts within the LCCCs; thus, lower compressive strength may be observed.

**Figure 21:**
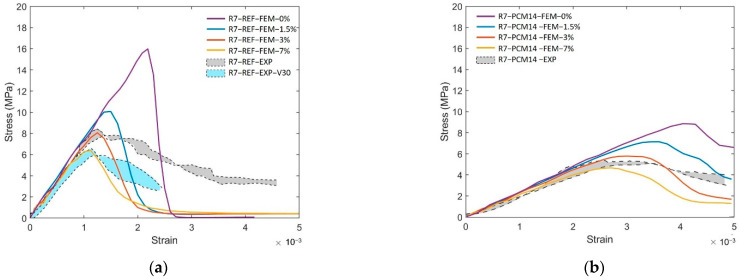
Comparison between experiment results and simulated results of (**a**) R7-REF specimens with different air content and vibration time and (**b**) R7-PCM14 specimens with different air content.

Therefore, in terms of the compression damage process of the LCCCs, the presence of the air voids is assumed to be a decisive factor. In order to elaborate the influence of air voids, R7 specimen with different air void fraction (by volume) was simulated and taken as an example. As can be seen in Figure 21a, the presence of air voids significantly decreased the compressive strength of R7-REF. In the simulated case with no air voids (R7-REF-FEM-0%), the compressive peak stress is substantially higher than the experimental results (hatched area marked as R7-REF-EXP). However, by increasing the air void fraction by only 1.5% percent, comparatively, the simulated peak stress immediately decreased to around 10 MPa, which is 37% lower than the case with no air voids. When increasing the air void fraction to 3%, the simulated curve (R7-REF-FEM-3%) has the best agreement with experiments within all used air void fraction values. A similar effect of the air voids on the compressive stress–strain curves can be also found in the PCM14 specimens. It is obvious in Figure 21b that the peak stress of the R7-PCM14 is significantly affected by the air content. Within all simulated curves, a specimen with 3% of air has the best agreement with experiment. Besides the stress–strain curves, the simulated crack pattern also shows good consistency with experiments (see Figure 20 as an example). Normally, diagonal cracks can be witnessed on the compressive fractured cementitious cubes due to the shear failure process. Comparatively, for the tested LCCCs in this study, diagonal cracks can be also observed, indicating the shear fracture of the LCCCs under compression. In addition to the diagonal cracks, due to the presence of air voids as well as the sharp corners along the pores of the cellular structure, vertical cracks can be also identified on the fractured specimen. This is then attributed to the tensile splitting fracture process similar to the example elaborated in Figure 19 and Figure 20.

The critical influence of air voids can be further demonstrated by creating a poorly vibrated mixture by decreasing the vibration time (R7-REF-EXP-V30). Vibration is very effective expelling air from fresh cementitious mixture [56]. Inversely, by decreasing vibration time from 60 to 30 s during the casting process, more air bubbles could be substantially maintained in the mixture; thus, the hardened specimens may possess higher air void fraction. Under this condition, it can be witnessed from Figure 21 that the peak stress also dropped to around 6MPa, which is lower than the properly vibrated specimens. Correspondingly, in the simulation cases, increasing the air content to 7% gives the best agreement with the poorly vibrated specimens(R7-REF-EXP-V30).

**Figure 22:**
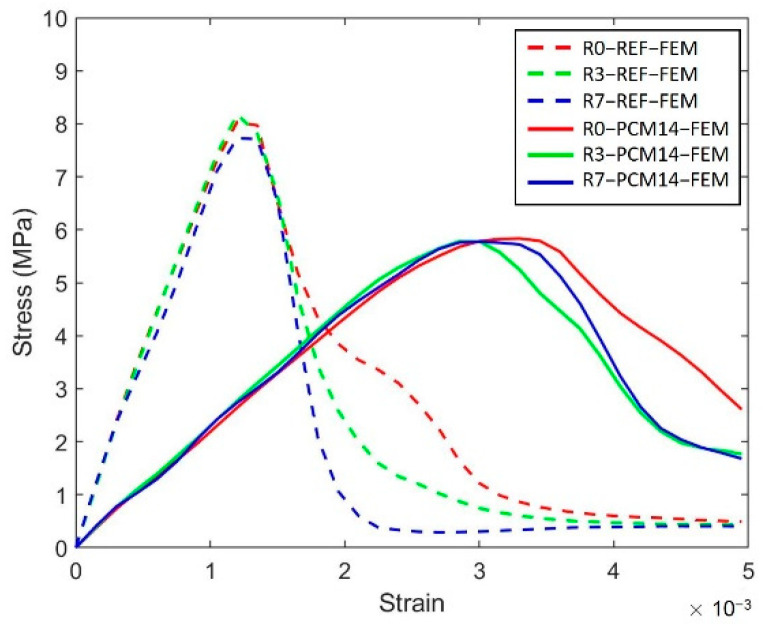
Simulated stress–strain curves of the LCCCs and 3% of air content are used for simulation.

It was previously observed that both for REF and PCM14, Voronoi specimens with higher randomness have a slightly lower compressive strength. While contradictory to the experimental observations, assuming the same fraction of introduced air content (3%) is contained, the simulated peak stress values of the LCCCs are almost the same (shown in Figure 22). Considering the compressive strength is rather sensitive to air void defects, this indicates that the entrapment of air bubbles more easily arises in the cellular specimens with higher randomness due to their complex structures.

**Figure 23:**
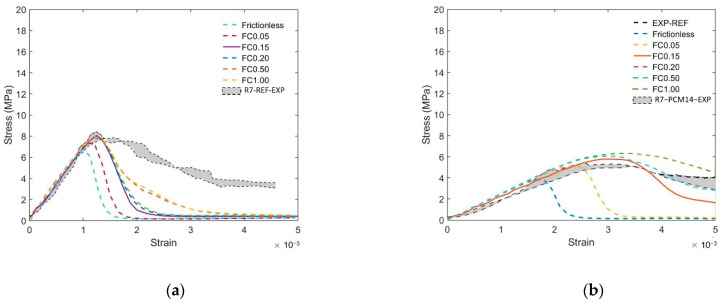
Influence of friction coefficient (FC) on the out-of-plane compressive behavior of (**a**) REF and (**b**) PCM14.

Still, a discrepancy can be found at post peak branch between the experimental results and the simulated curve: the experimental results show higher toughness compared to the simulated curve. According to the principles of concrete fracture mechanics [45], a possible reason might be that the friction between the loading plate still affects the post-peak part of the stress–strain curve even when plastic films were used in the experiment in the out-of-plane condition. Therefore, the influence of friction coefficient (FC) on the compressive behavior in the out-of-plane direction was also simulated. As can be seen from Figure 23, increasing the FC from 0.15 to 1.00, the area below the stress–strain curves of the R7 specimen substantially increased, while without visible change to the peak stress (see Figure 23). By contrast, decreasing the FC from 0.15 to the frictionless condition (FC = 0), the simulated out-of-plane strength of the REF (Figure 23a) dropped by approximately 20%. Similar trend can also be witnessed on the compressive behavior of the PCM14 specimens (Figure 23b). The phenomenon that the LCCCs exhibit higher compressive strength under larger FC was mainly caused by the confining effect generated at the interface between the loading plates and the cementitious specimen. This boundary confining effect is commonly seen in cementitious materials under uniaxial compression [45,57]. It was reported in [57] that the confinement induced by friction obviously increased concrete compressive strength due to the horizontal frictional force confining the tensile stress development between micro-defects of concrete. In this sense, a higher compressive load is required to rupture the specimen. For the studied specimen in this work, it seems that when the FC increased to 0.15, the limit of the horizontal frictional force was reached. The peak stress was not able to further increase even when the FC was elevated to a higher value as a result. What should be noted is that, in the simulations where the friction coefficient was set to be 0.15, the peak stress of the simulated curves is higher than that in the frictionless condition due to the increased restraint. As a result, the actual void defects content of the specimen may be slightly lower than the introduced value. Then, the actual void defects content of the tested LCCCs is conjectured within a range from 1.5% to 3% by volume of the entire cubic domain or from 0.75% to 1.5% by volume of the LCCCs specimens.

Apart from the compressive strength, hither boundary friction also gave LCCCs a larger softening tail. This phenomenon can be explained by the energy release mechanism during the fracture process of cementitious materials [57]. Under the frictionless condition, cracks chose the weakest path of the specimens, thus releasing the lowest amount of energy. However, when the higher friction is present, the cracking has to avoid the confined region, thus releasing more energy. This reflected on the stress–strain curves by a longer and larger softening post-peak branch. Still, even increasing the FC to 1.00, the softening branch of experimental stress–strain curves has a larger area. Another possible reason might be that the introduced air voids did not completely cover the air size distribution in the experiment: smaller air voids are able to induce local fracture which generate more distributed cracks making the experimental LCCCs show “softer” behavior in the post-peak branch.

### 4.5. In-Plane Compressive Behavior

Although the in-plane direction of the LCCCs is supposed to be used for insulating heat transfer—namely, it should not be exposed to external load—the in-plane compressive behavior is still worth the comparison to the out-of-plane damage behavior to understand the influential factors.

**Figure 24:**
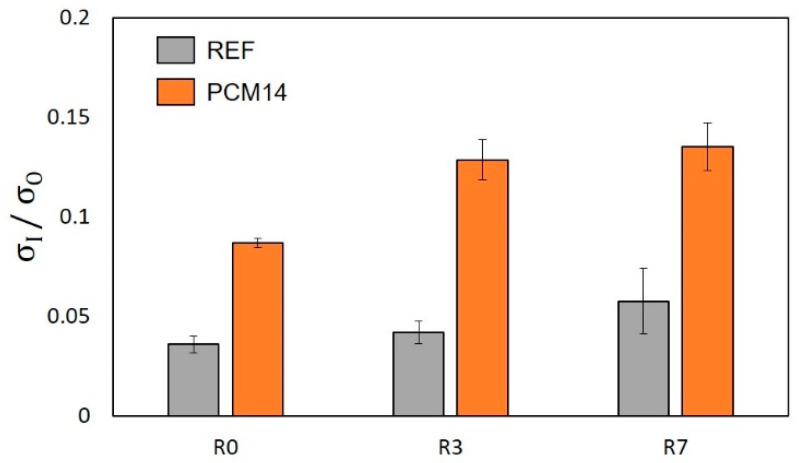
Ratio of the in-plane strength (σ_I_) to out-of-plane strength (σ_O_) of the LCCCs.

**Figure 25:**
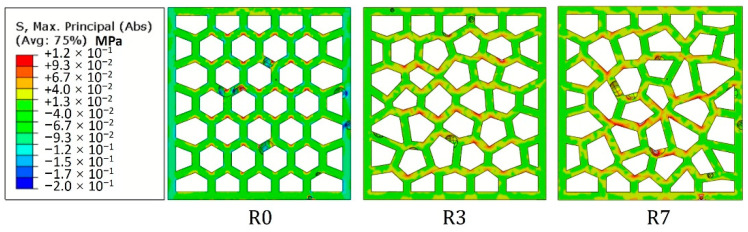
Stress distribution of the LCCCs within the elastic regime.

**Figure 26:**
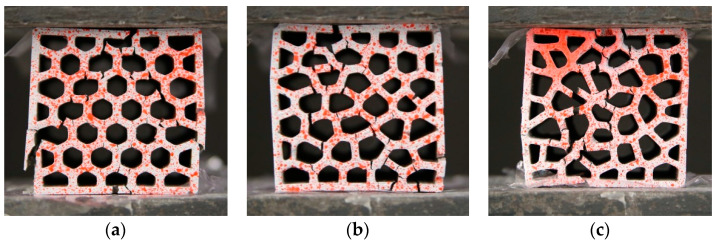
Crack pattern of the LCCCs, (**a**) R0, (**b**) R3 and (**c**) R7.

**Figure 27:**
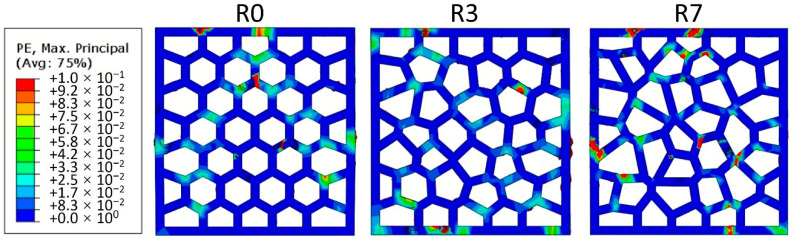
Simulated damage pattern of the LCCCs, plastic strain is indicated.

Figure 24 shows the ratio between the in-plane compressive strength (*σ*_I_) to the out-of-plane compressive strength (*σ*_O_). In general, the in-plane strength of the LCCCs decreases dramatically compared to the out-of-plane direction. This was mainly caused by the presence of tensile stress on the plate elements of the LCCCs when loaded from the in-plane direction. It is clearly shown in Figure 25 that tensile stress concentrates at the sharp corners of the cellular structure. As a result, cracks generated and propagated from these locations eventually lead to the failure of the specimen. Distributed cracks on the plate elements of the LCCCs can be found in Figure 26. Correspondingly, it can also be seen that similar damage pattern can also be observed from the simulation results in Figure 27: the locations of distributed plastic strain are present on the specimens.

The influence of Voronoi randomness and mPCM on the in-plane strength is also visible in Figure 24. The difference between in-plane and out-of-plane strength decreases with respect to the Voronoi randomness; moreover, the mPCM incorporated LCCCs (R7-PCM14) has the least difference between its in-plane and out-plane compressive strength.

**Figure 28:**
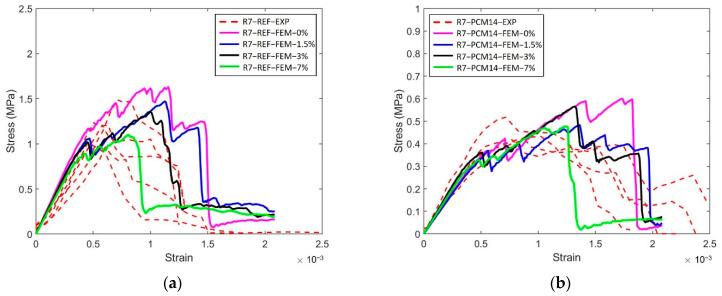
In-plane stress–strain curves of (**a**) R7-REF and (**b**) R7-PCM14; note that the y-axes of the two figures are different.

The influence of air voids defects is studied by comparing the experimental results and numerical simulations on R7 specimens. It is shown in Figure 28 that for both types LCCCs made by REF and PCM14, introducing air voids also decreases the in-plane peak stress, while the negative effect is much more moderate compared to the out-of-plane direction. This was due to the tensile stress distribution on the plate elements of the LCCCs. Even without air voids, the sharp corners already contribute as a critical part on the specimens. As a result, the influence of air voids defects is considerably mitigated.

**Figure 29:**
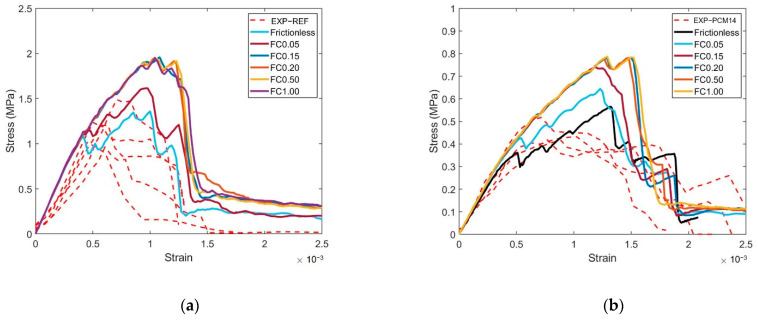
Influence of the friction coefficient (FC) on the simulated in-plane stress–strain curves of (**a**) R7-REF and (**b**) R7-PCM14; note that the y-axes of the two figures are different.

Similar to the out-of-plane direction, the influence of friction coefficient is also investigated for the in-plane direction. It can be seen in Figure 29, when the friction coefficient is increased from frictionless condition (FC = 0) to 0.15, an obvious increase in the in-plane compressive strength can be observed, both for the REF (Figure 29a) and PCM14 (Figure 29b) specimens. As explained previously, the horizontal frictional force introduced at the interface between the specimen and the loading plates generated restraining effect; thus, higher stress is required to rupture the specimen [57]. When FC reached 0.15, the limit of the horizontal frictional force was reached, and the stress could not increase further. It was found in the previous section that owing to this limit, in the out-of-plane direction, assigning FC = 0.15 has given the simulation result better agreement with experimental results. However, at the in-plane direction, the simulated curve has relatively higher strength when 0.15 was adopted as the FC value. Thus, it is assumed that during the experiment, there was less influence from the frictional force on the stress–strain response at the in-plane direction. This might indicate that, at the in-plane direction, the contact between specimen surface and the loading plates may not correspond to a simple surface-to-surface penalty contact algorithm. Elastic slide or a shear limit may also exist at the interface, which makes the contact between the specimen and loading plates closer to a frictionless condition. However, this was out of the main scope of this work, and it can be investigated in future studies.

## 5. Conclusions

This work aims to fabricate a novel lightweight cementitious cellular composite (LCCCs) incorporated with micro-encapsulated phase change materials (mPCM). Mechanical properties of the LCCCs were investigated by experiments and numerical simulations. With the aid of a 3D printing technique, LCCCs with Voronoi structures were prepared using a reference mortar and mPCM incorporated mortar. The damage mechanism of the LCCCs was clarified. The influence of Voronoi randomness and mPCM on the compressive behavior of the LCCCs were elaborated on. According to the obtained results, several conclusions can be drawn as follow:

- The highly randomized Voronoi structure is found to have similar mechanical properties to a regular honeycomb. Considering its known better thermal properties, the highly randomized Voronoi structure is preferable rather than traditional regular honeycomb for the LCCCs.
- In contrast to conventional cementitious materials, incorporating mPCM with the LCCCs does not obviously decrease their compressive strength. This indicates that the strategy of combining mPCM with highly randomized Voronoi structures has great potential to fabricate cementitious lightweight composites with phase change capacity.
- The numerical model is experimentally calibrated in terms of the constituent material behavior. The simulation results of the compressive behavior of the LCCCs show very good agreement with the experiment.
- An additional air void defect was found to be a determinative factor on the compressive strength of the LCCCs. Stress intensity caused by the air voids defects leads to potential cracking, which initiates the fracture of the LCCCs and substantially decreases the compressive strength.

In general, the LCCCs show potential to be used for thermal insulation purpose. Still, the properties of the LCCCs can be further improved and need more future work. In terms of mechanical property, as indicated in this work, the presence air voids drastically decrease the compressive strength. Therefore, in future studies, optimizing the cementitious mixture and the cellular structure such that less air voids are entrapped could be a direction to further improve the compressive strength. In addition, for larger scale construction, direct 3D printing of this type of materials would be more preferable, and it is also worth investigating in future studies.

## Figures and Tables

**Table 1:** Mixture proportions and corresponding slump flow values.

No.	CEMI 42.5 N (g/L)	Fly Ash (g/L)	Sand (g/L)	SP (Glenium 51) (g/L)	mPCM (g/L)	Water (g/L)	w/b	mPCM Volume Ratio (%)	Slump Flow (mm)
REF	510.2	602.7	510.2	1.9	0	366.4	0.33	0	243.0
PCM14	421.8	498.5	295.2	3.1	126.5	379.6	0.41	14.4	243.3

**Table 2:** Specimen characteristics.

Randomness of Specimen	Porosity	Cell Wall Thickness (mm)
0	0.498	2 (for H20 is 1)
0.3	0.493	2 (for H20 is 1)
0.7	0.484	2 (for H20 is 1)

**Table 3:** Compressive input parameters of REF.

Yield Stress (MPa)	Inelastic Strain	Damage Parameter
30.4	0	0
33.6	0.001115	0
14.4	0.013047	0.571429
7.68	0.020946	0.771429
3.44	0.025923	0.897619
0.88	0.037988	0.973810

**Table 4:** Tensile input parameters of REF.

Yield Stress (MPa)	Cracking Strain	Damage Parameter
5.25	0	0
3.00	0.000172	0.428571
1.50	0.000417	0.714286
0.45	0.000688	0.914286
0.15	0.000808	0.971429

**Table 5:** Compressive input parameters of PCM.

Yield Stress (MPa)	Inelastic Strain	Damage Parameter
11.2	0	0
16.8	0.000264	0
7.0	0.014235	0.583333
3.5	0.026140	0.791667
1.4	0.032141	0.916667
0.3	0.048684	0.982143

**Table 6:** Tensile input parameters of PCM.

Yield Stress (MPa)	Cracking Strain	Damage Parameter
1.8375	0	0
1.0500	0.000237	0.428571
0.5250	0.000516	0.714286
0.1575	0.000830	0.914286
0.0525	0.000971	0.971429

**Table 7:** Model input plasticity parameters.

Input Parameters	Value
Dilation angle (°)	35
Eccentricity	0.1
fb_0_/fc_0_	1.16
K	0.667
Viscosity parameter	0.001

## Data Availability

Data will be made available upon request.
